# Impact of Oral Treatment on Physical Function in Older Patients Hospitalized for Heart Failure: A Randomized Clinical Trial

**DOI:** 10.1371/journal.pone.0167933

**Published:** 2016-12-13

**Authors:** Kazutaka Ueda, Masashi Kasao, Motoaki Shimamura, Hironori Haruta, Shuya Nitta, Mitsunobu Kaneko, Yukari Uemura, Hiroyuki Morita, Issei Komuro, Tetsuro Shirai

**Affiliations:** 1 Department of Cardiology, Tokyo Metropolitan Police Hospital, Tokyo, Japan; 2 Department of Cardiovascular Medicine, the University of Tokyo Hospital, Tokyo, Japan; 3 Biostatistics Division, Central Coordinating Unit, Clinical Research Support Center, the University of Tokyo Hospital, Tokyo, Japan; Tokai University, JAPAN

## Abstract

**Background:**

Frailty is a characteristic of older patients with heart failure, who undergo functional decline during hospitalization. At present, continuous intravenous infusion of diuretics is widely used for the treatment of hospitalized patients with heart failure. In this prospective, randomized, open-label controlled trial, we tested whether an early switch from continuous intravenous infusion therapy to oral treatment with diuretics prevents functional decline in patients hospitalized for heart failure.

**Methods:**

A total of 59 patients hospitalized for heart failure were randomized to either continuous intravenous infusion (n = 30) or oral medication (n = 29) within 48 h of admission. The primary outcome was the Barthel index, a universally utilized scale to assess the functional status of patients in their activities of daily living, assessed at 10 days. Secondary outcomes included the number of daily steps counted using pedometers and average hospital costs.

**Results:**

Barthel index scores were significantly higher in the oral medication group than in the intravenous group (78.1 ± 20.8 vs. 59.6 ± 34.2, *P* = 0.029). The number of daily steps was significantly higher in the oral treatment group relative to the intravenous group (*P* < 0.001), and the average hospital costs were similar between the randomized groups. Multivariate analysis revealed that oral medication was a significant independent predictor of Barthel index score at day 10, and the number of daily steps was significantly associated with the patient’s functional outcome.

**Conclusions:**

This trial showed that, in patients hospitalized for heart failure, oral medication increased functional independence during hospitalization compared with sustained continuous intravenous infusion, most likely because the release from the infusion line enabled the patients to be more mobile. Notably, these beneficial effects were achieved without increasing hospital costs.

## Introduction

Heart failure (HF) is a major cause of hospitalization in patients ≥ 65 years old in developed countries [1]. These older patients frequently exhibit frailty, which is a biological syndrome that reflects a state of decreased physiological reserve and vulnerability to stressors such as acute or chronic illness and hospitalization [2]. Fifty-one percent of hospitalized patients with HF > 65 years old reportedly exhibit frailty, which is much more frequent than the 10% of community-dwelling older adults [3]. Frail patients are at risk of physical and/or cognitive decline during hospitalization, and are more likely to have impaired activities of daily living (ADL), increased risk of re-hospitalization, decreased quality of life (QOL), and increased mortality [4]. These adverse events also increase health care costs. Therefore, novel interventions are needed to improve outcomes in frail elderly patients with cardiovascular diseases, especially in aging populations.

Although intravenous (IV) loop diuretics are an essential component of current treatment and are administered to approximately 90% of patients hospitalized with HF [5], this infusion route may restrict mobility and lead to excessive bed rest, which reportedly contributes to functional decline [6]. Oral medication (OM), in contrast, may preserve patient mobility during hospitalization. However, no reports have described the effects of different administration routes on functional decline in patients with HF.

This prospective controlled study, therefore, investigated whether an early switch from continuous IV infusion to OMs, such as tolvaptan, a selective oral vasopressin V2 receptor antagonist, prevents functional decline in patients hospitalized for HF.

## Methods

### Participants

Hospitalized patients were eligible for enrollment if they were ≥ 20 years of age and had presented within the previous 24 hours with acute decompensated HF or exacerbation of chronic HF from any cause, diagnosed on the basis of the presence of at least one symptom (dyspnea, orthopnea, or edema) and one sign (rales, peripheral edema, ascites, or pulmonary vascular congestion on chest radiography) of HF. There was no pre-specified inclusion criterion with respect to ejection fraction. The exclusion criteria included acute myocardial infarction at the time of hospitalization, hemofiltration or dialysis, and systolic arterial blood pressure < 90 mmHg. Patients who needed continuous fluid replacement and/or inotropic agents were also excluded. All patients provided written informed consent before enrollment. The study protocol complied with the Declaration of Helsinki, and The Ethics Committee of the Tokyo Metropolitan Police Hospital approved the study protocol on December 20, 2013 (S1 File), and the first patient was recruited on January 11, 2014. The recruitment ended in February, 2015, and the follow-up was completed in March, 2015. The trial was registered with the UMIN Clinical Trials Registry (UMIN 000013091 https://upload.umin.ac.jp/cgi-open-bin/ctr_e/ctr_view.cgi?recptno=R000015276). The registration was disclosed after patient recruitment began, simply due to the delay of entry process. The authors confirm that all ongoing and related trials for this drug/intervention are registered.

### Study Design

This open-label, prospective, randomized study design was conducted at Tokyo Metropolitan Police Hospital in Tokyo between January 2014 and March 2015. Patients were initially treated with continuous peripheral IV infusion of a standard HF drug, such as loop diuretics. On the next day (day 1) or day 2 of admission, eligible patients were randomized in a 1:1 ratio to either continuous IV infusion (IV group) or OM (OM group) using computer-generated permuted blocks managed by an independent study center. The participants in the IV group underwent continuous IV infusion, and the participants in the OM group were switched to OM. All participants were advised by the physicians to minimize bed rest and were provided with salt-restricted meals (containing 6 g of salt daily). Fluid intake was restricted to < 1000 ml daily except for the patients for whom fluid restriction was lifted during tolvaptan usage, according to its prescribing information. The IV group received continuous IV infusion of drugs, such as furosemide and natriuretic peptide (carperitide), accompanied by 500 ml per day of replacement fluid (5% dextrose or maintenance fluid, including 2.7% glucose, 25 mEq Na^+^Cl^-^, and 10 mEq K^+^). All patients received standard HF therapy, including diuretics, digoxin, angiotensin-converting enzyme inhibitors, angiotensin II receptor blockers, β-blockers, aldosterone blockers, hydralazine, and/or nitrates, and non-drug treatment, such as bladder catheterization and cardiac rehabilitation, all of which were administered at the discretion of the treating physician. For safety reasons, the continuous IV infusion was administered in the OM group at the treating physicians’ discretion for patients who showed dehydration or inadequate HF improvement. On the basis of the intention-to-treat principle, all participants allocated to each group were analyzed. Patients and physicians were made aware of the treatment assignments; however, imaging analysis, rehabilitation, and social work were performed by healthcare professionals who were blinded to the study details.

### Primary End Point

To evaluate functional status, the Barthel index (BI) was assessed as the primary end point at day 10, or on the last day before discharge, if the patient was discharged earlier than day 10. The 10 items within the scale include feeding, grooming, bathing, dressing, bowel and bladder care, toilet use, ambulation, transfers, and stair-climbing. Each performance item is rated using points, with the sum of all items indicating the patient’s ADL (0–100 points) [7]. The maximum possible score is 100, which indicates that the patient is fully independent in physical functioning. The lowest score is 0, which represents a completely dependent and bedridden state.

### Secondary End Points

The secondary end points included the Functional Independence Measure (FIM) and its subscales, which analyze both motor and cognitive functions. Health-related QOL was assessed using the 36-Item Short-Form Health Survey (SF-36 version 2 in Japanese, licensed by iHOPE International, Tokyo, Japan). Daily steps were counted by pedometers until day 10 of the hospital stay. The length of hospital stay, admission costs, and patient discharge location were also assessed. Information regarding the patients’ functional status and overall health was obtained by interviewing the patient and/or an accompanying family member. The participants’ functional status and overall health before the onset of the illness that led to hospitalization were also assessed as baseline data. All interviews were conducted by well-trained nurses who followed a written protocol to standardize the data. To assess the dose of furosemide, other loop diuretics, such as 30 mg of azosemide or 4 mg of torasemide, were considered to be equivalent to 20 mg furosemide.

### Sample Size Calculation

This study was powered to detect a BI score change of 20 [8]. A sample size of 52 participants (26 per group) provided 80% power to detect a change with an SD difference of 25 when using a two-sided alpha value of 0.05.

### Statistical Analysis

Continuous variables are presented as the mean ± SD, and skewed variables are presented as the median with the interquartile range, as appropriate. The two groups were compared using the Student’s t-test, and comparisons among multiple groups were performed by ANOVA, followed by Tukey-Kramer honest significant difference (HSD) analysis. Categorical variables were compared using the Fisher’s exact or the chi-square test, and are presented as the frequency (percentage). Steps measured using pedometers were compared with the Wilcoxon test. Differences in BI scores for the number of daily steps were tested using the *P* for the trend obtained from the ANOVA analysis. The correlation between BI scores and number of daily steps was also analyzed using Spearman test. Multivariate analysis was used to assess the independent risk of drug administration route on the clinical end point. Candidate variables, including allocated group, age, BI and B-type natriuretic peptide (BNP) at baseline, and days of bladder catheterization, were selected on the basis of variables reported in the literature and were incorporated into the univariate and multivariate analysis. The univariate and multivariate analysis data are presented as β ± SE. Analyses were performed using JMP (version 11.0). All tests of significance were two-tailed, and *P* values < 0.05 were considered statistically significant.

## Results

### Baseline Characteristics

**Fig 1:**
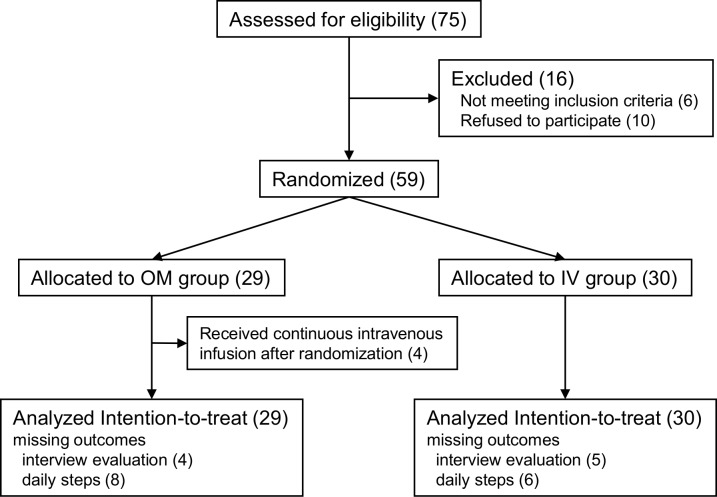
CONSORT (Consolidated Standards of Reporting Trials) flow chart of the study participants.

A total of 75 patients were referred for participation, and 59 patients were randomly assigned to the OM (n = 29) or IV (n = 30) group between January 2014 and February 2015 (Fig 1). The clinical characteristics of the two treatment groups, including age, sex, BI, and FIM score, were similar (Table 1); the BI, FIM, and SF-36 scores at baseline showed the participants’ functional status and overall health before onset of the illness that led to hospitalization. There were no differences in the parameters indicating HF severity, including the New York Heart Association (NYHA) symptom class, estimated right ventricular systolic pressure, and serum BNP level at baseline (Tables 1 and 2). Four patients in the OM group underwent continuous IV infusion during the intervention period (three for dehydration and one for worsening HF); all were analyzed by intention to treat. Some patients in both groups were not included in the evaluation of BI, FIM, and SF-36 because of declination of the interview. Daily step counts were not available for some patients in both groups due to an unexpected measurement problem. There were no differences in the mean doses of diuretics received in the pre-randomization period (Table 1).

**Table 1:** Clinical characteristics

	OM group	IV group	
	(n = 29)	(n = 30)	p value
Age, y	82.1±10.2	82.6±9.5	0.83
Age ≥65 y, n (%)	28(97)	28(93)	1.00
Male, n (%)	14 (48)	15 (50)	1.00
NYHA classification III-IV, n (%)	25 (86)	26 (87)	1.00
sBP, mmHg	119.4±24.5	118.3±20.2	0.85
dBP, mmHg	70.9±17.6	66.0±14.5	0.26
HR, rpm	84.3±24.2	86.5±23.2	0.72
plasma/serum variables			
Creatinine, μmol/L	1.1±0.6	1.2±0.6	0.43
eGFR ml/min/1.73mm2	50.5±18.2	45.9±20.0	0.37
Hemoglobin, g/dl	12.4±1.6	11.6±2.5	0.16
BNP, pg/ml	779±1214	715±750	0.81
Ejection Fraction, %	53.1±18.1	54.9±20.0	0.74
eRVPs, mmHg	44±11	44±13	0.95
Etiology of HF			
Ischemia heart disease, n (%)	5 (17)	6 (20)	1.00
Cardiomyopathy, n (%)	4 (14)	2 (7)	0.64
Valvular disease, n (%)	8 (28)	7 (23)	0.94
Hypertensive heart disease, n (%)	9 (31)	7 (23)	0.71
Others, n (%)	2(7)	8(27)	0.09
Comorbidity			
Hypertension, n (%)	12 (41)	13 (43)	1.00
Diabetes mellitus, n (%)	8 (28)	7 (23)	0.94
Hyperlipidemia, n (%)	4 (14)	5 (17)	1.00
Chronic kidney disease, n (%)	6 (21)	9 (30)	0.60
Atrial fibrillation, n (%)	18 (62)	15 (50)	0.50
Dose of IV diuretics prior to randomization			
Furosemide, mg/day	28.4±9.6	27.0±8.1	0.59
Natriuretic peptide (carperitide), μg/day	1828±539	1800±610	0.86

Values are mean ± SD or n (%). NYHA indicates New York Heart Association; sBP, systolic blood pressure; dBP, diastolic blood pressure; HR, heart rate; eGFR, estimated glomerular filtration rate; BNP, B-type natriuretic peptide; eRVPs, estimated right ventricular systolic pressure; HF, heart failure; IV, intravenous.

**Table 2:** Comparison of primary and secondary outcomes between OM group and IV group

	OM	IV	OM	vs IV	
	group	group	(95	%CI)	p value
Primary Outcome	(n = 25)	(n = 25)			
BI					
baseline	92.6±10.8	92.2±14.9	0.4	(-7.0 to 7.8)	0.91
day10	78.1±20.8	59.6±34.2	18.6	(1.7 to 35.4)	0.03
Secondary Outcomes					
Interview Evaluation	(n = 25)	(n = 25)			
FIM (total)					
baseline	115.0±11.0	112.6±15.7	2.4	(-5.3 to 10.2)	0.53
day10	106.0±21.8	88.2±32.4	17.7	(1.4 to 34.1)	0.03
FIM (motor)					
baseline	86.1±5.7	83.6±11.1	2.6	(-2.5 to 7.6)	0.31
day10	76.4±17.6	61.4±24.3	15.0	(2.4 to 27.5)	0.02
FIM (cognition)					
baseline	29.6±6.3	29.0±6.3	0.6	(-3.0 to 4.1)	0.75
day10	29.7±6.3	25.7±8.2	4.0	(-0.3 to 8.3)	0.07
SF-36					
baseline	108.4±26.5	104.2±26.7	4.2	(-10.9 to 19.4)	0.58
day10	100.7±21.0	92.0±25.3	8.8	(-4.5 to 22.0)	0.19
Pedometer measurement	(n = 21)	(n = 24)			
Steps, /day	313 (128, 779)	113 (50, 231)			<0.001
Efficacy, Time, Cost, Place, Others	(n = 29)	(n = 30)			
BW, kg					
baseline	54.6±15.3	51.3±11.7	3.3	(-4.2 to 10.8)	0.70
day5	51.5±14.4	49.4±11.4	2.1	(-5.0 to 9.2)	0.92
day10	49.8±14.1	48.3±11.4	1.5	(-5.5 to 8.5)	0.95
BNP, pg/ml					
baseline	779±1214	715±750	64.2	(-482 to 610)	0.81
day5	542±724	632±1064	-89.8	(-585 to 405)	0.72
day10	309±265	475±613	-166.6	(-420 to 87)	0.21
eRVPs, mmHg					
baseline	44±11	44±13	0.2	(-6.6 to 7.0)	0.95
day10	38±10	37±14	1.8	(-5.1 to 8.6)	0.61
Continuous intravenous infusion					
time, days	2.6±1.6	9.8±7.2	-7.2	(-10.0 to -4.4)	<0.001
Bladder catheterization time, days	5.3±3.8	6.4±6.5	-1.2	(-4.0 to 1.6)	0.40
Intervention of rehabilitation, n (%)	14 (48)	16 (53)			0.90
Hospitalization time, days	17.5±7.6	18.6±12.3	-1.1	(-6.6 to 4.4)	0.69
Hospitalization cost, x1000yen	737±360	839±530	-101	(-359 to 158)	0.44
Place of discharge					
Private home, n (%)	26 (90)	26 (87)			1.00
Long-term care institution, n (%)	3 (10)	3 (10)			1.00
Death, n (%)	0 (0)	1 (3)			1.00

Steps is shown as median (first, third quartile, respectively), and the other values are mean ± SD or n (%). CI indicates confidence interval; BI, Barthel Index; FIM, Functional Independence Measure; SF-36, 36-item Short-Form General Health Survey; BW, body weight; BNP, B-type natriuretic peptide; eRVPs, estimated right ventricular systolic pressure.

### Primary Outcome

**Fig 2:**
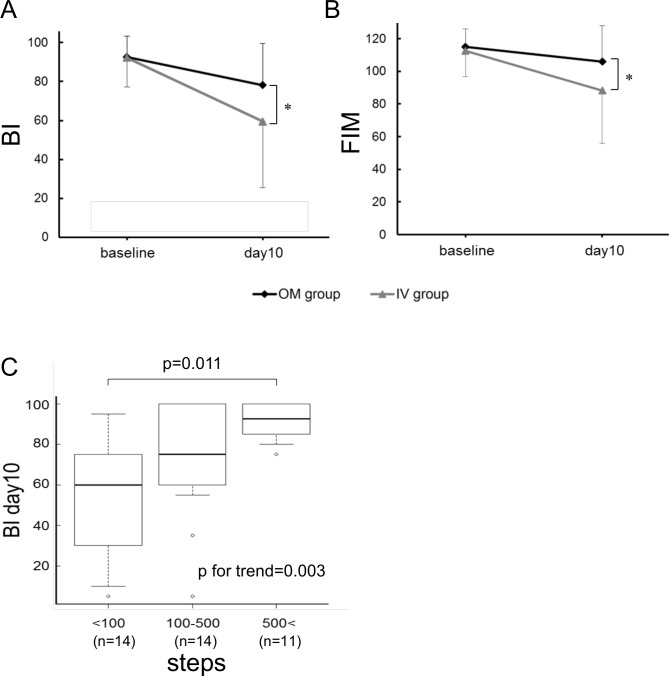
Changes in activities of daily living (ADL) scores after 10 days of hospitalization, and BI scores based on the number of daily steps. The Barthel Index (BI, A) and Functional Independence Measure (FIM, B) scores in the OM and IV groups are shown; *P < 0.05. The BI score at day 10 according to the number of daily steps is shown (C).

To evaluate whether an early switch to OM would prevent functional decline in patients hospitalized for HF, the BI score was assessed 10 days after admission, or on the last day before discharge, if the patient was discharged earlier than day 10. There was no significant difference in the mean duration until assessment between the two groups; thus, the day of assessment is expressed here simply as day 10. The day 10 BI score was significantly higher in the OM group than in the IV group (78.1 ± 20.8 vs. 59.6 ± 34.2, respectively, *P* = 0.029; Table 2, Fig 2A and S1 Fig). Among the individual components of BI, significantly higher scores in each component of transfers (bed to chair), grooming, bowels, and bladder were observed at day 10 in the OM group than in the IV group (S1 Table).

### Secondary Outcomes

Secondary outcomes, including FIM and SF-36 scores, number of steps, costs, and time and place of discharge, are shown in Table 2. Consistent with the primary outcome, the total FIM score at day 10 was significantly higher in the OM group than in the IV group (106.0 ± 21.8 vs. 88.2 ± 32.4, respectively, *P* = 0.032; Table 2, Fig 2B and S1 Fig). The FIM was grouped into two subscales: motor function and cognitive function. The score of motor function was significantly higher in the OM group than in the IV group (76.4 ± 17.6 vs. 61.4 ± 24.3, respectively, *P* = 0.019; Table 2 and S1 Fig). There was no difference in the QOL score, as evaluated by SF-36, between the groups. The number of daily steps (as counted by pedometers), which was used to assess the level of activity during hospitalization, was significantly higher in the OM group relative to the IV group (*P* < 0.001; Table 2). Because an unexpected measurement problem occurred in the first 14 consecutive patients (OM group, 8; IV group, 6), the pedometer data from these patients were excluded from the analysis.

All patients in the OM group and one patient in the IV group were treated with the oral diuretic agent tolvaptan. The mean durations of continuous IV infusion in the OM group and IV group were 2.6 days and 9.8 days, respectively (*P* < 0.001). The average days and costs of hospitalization were not significantly different (Table 2 and S1 Fig). Additionally, there was no difference in the place of discharge; three patients (10%) in each group were discharged to long-term care institutions for continuous rehabilitation (Table 2).

## Effects, Adverse Events, and Safety

The effects of oral and continuous infusion therapy on HF were compared; there were no significant differences in body weight, BNP, or right ventricular systolic pressure (as estimated by echocardiography) at baseline and at each time point (Table 2). In addition, there was no difference in the average number of days of patients requiring bladder catheterization or in the rates of referral to the rehabilitation program. One IV group patient died at day 15 because of severe cardiomyopathy leading to lethal arrhythmia, but in-hospital mortality was not significantly different between the groups. No other severe adverse effect was observed. The average serum sodium level was significantly higher in the OM group than in the IV group (142 ± 3 vs. 137 ± 3 mEq/L at day 5, respectively, *P* < 0.001; and 140 ± 3 vs. 136 ± 4 mEq/L at day 10, respectively, *P* < 0.001), presumably because of differences in tolvaptan usage, although no significant symptomatic effect due to abnormal sodium levels was found. The total dose of IV diuretics received after randomization was significantly lower in the OM group, but the use of oral furosemide equivalents and tolvaptan was significantly increased (Table 3).

**Table 3:** Dosage of Diuretics

	OM group	IV group	
	(n = 29)	(n = 30)	p value
Intravenous Infusion			
Furosemide, mg	34±62	194±149	<0.001
Natriuretic peptide (carperitide), μg	2310±3371	9400±6441	<0.001
Oral medications			
Furosemide or furosemide equivalent, mg	284±169	170±149	0.01
Thiazide diuretics (Torasemide), mg	1.4±4.0	1.6±4.1	0.84
Aldosterone-blocker (Spironolactone), mg	61±95	38±96	0.36
Tolvaptan, mg	44±22	0.3±1.4	<0.001

This table represents the total dosage of diuretics after randomization.

### Predictors of Patient Function After 10 Days of Hospitalization

The BI score at day 10 correlated significantly with group allocation and days of bladder catheterization in both the univariate and multivariate analyses, and with BNP at baseline in the multivariate analysis only. Multivariate analysis showed that allocation to the OM group resulted in an increase in the BI score at day 10 by 15.3 points relative to the IV group, but the score at baseline was not associated with outcome (Table 4).

**Table 4:** Predictors of BI score at day10 in patients hospitalized for heart failure

		Unavailable Analysis			MultivariableAnalysis	
	β	SE	p value	β	SE	p value
OM group	18.56	8.21	0.03	15.26	6.06	0.02
Age, y	-1.30	0.42	<0.01	-0.68	0.34	0.06
BI baseline per unit	0.68	0.34	0.05	0.41	0.26	0.11
BNP baseline per 100pg/ml increase	-0.68	0.42	0.11	-0.71	0.30	0.02
Bladder catheterization time, days	-4.00	0.81	<0.001	-3.27	0.73	<0.001

This table represents the results of both univariate and multivariate regression analysis. BI indicates Barthel index; BNP, B-type natriuretic peptide.

As shown in Table 2, because the number of daily steps was significantly higher in the OM group, we evaluated whether the effects of group allocation on functional outcome were mediated by the number of daily steps during hospitalization. Daily steps were positively correlated with the BI score at day 10 (Spearman ρ = 0.550; *P* < 0.01). As shown in Fig 2C, a higher BI score at day 10 was significantly correlated with an increase in the median number of daily steps (*P* for trend = 0.003). The mean BI score was significantly higher for the patients who walked > 500 steps than for those who walked < 100 steps (*P* = 0.011). Moreover, multivariate analysis showed that this classification of daily steps (< 100, 100–500, and > 500) was a significant independent factor associated with BI score at day 10 (β = 14.38 ± 5.01, *P* = 0.007).

## Discussion

This randomized trial showed that, in patients hospitalized for HF, an early switch from continuous IV infusion to OM compared with a continuation of IV infusion significantly prevented functional decline during hospitalization. In addition, the early switch to OM led to an increase in the number daily steps, most likely because release from the infusion line gave the patients unrestricted mobility. Notably, these benefits were achieved without increasing costs. In the aging of population, functional outcomes are especially important in patients who are frail and at high risk for functional decline and institutionalization for long-term care. Clinical intervention programs such as ours may improve patient outcomes.

Although there has been some overall improvement in the survival of patients with HF over the past two decades, mortality and re-hospitalization rates in elderly patients with HF have not substantially improved [9, 10]. This lack of progress in improving outcomes in older patients with HF may be due to the fact that most studies have not taken into account frailty, a common and key characteristic of elderly patients with HF. It has only very recently been reported that limitations in ADL and cognitive function were strongly associated with mortality in older patients hospitalized with HF [11–13]. Consequently, prevention of functional decline during hospitalization with HF is a critical theme; nevertheless, only a few studies have assessed factors that affect this adverse event. Cardiac rehabilitation is one potential intervention for improving the overall health of frail patients, although its efficacy has yet to be fully proven [2].

Advances and innovations in therapies for decompensated HF have allowed physicians to select optimal medication strategies, especially for mild to moderately severe HF. Our study showed that an early switch from continuous IV infusion to OM was significantly associated with the preservation of physical activity, leading to the prevention of functional decline. Bed rest is seldom recommended for treating HF, except in the very severe stage of the disease, because it causes a substantial loss of skeletal muscle in older adults, particularly in the lower extremities. Limiting the use of devices that restrict mobility, such as infusion lines and urinary catheters, may help prevent functional decline in hospitalized older patients [14]. Additionally, we found no differences in the effects of HF therapy between the groups on body weight, plasma BNP levels, and estimated right ventricular systolic pressure, which suggests that oral therapy is not necessarily inferior to IV infusion therapy for mild to moderate HF. This is consistent with findings from a previous study [15].

Tolvaptan promotes water excretion without altering renal hemodynamics or sodium and potassium excretion and has been used in the treatment of hypervolemic and euvolemic hyponatremia in the United States. This oral drug has been used to reduce volume retention in HF patients in Japan, and its efficacy and safety is reported to be comparable with IV diuretics. Tolvaptan is also known to preserve or increase serum sodium levels at day 10 in HF patients [16]. Consistent with this, a higher serum sodium level was observed in the OM group, in which tolvaptan was used for all patients. Although better clinical outcomes have been reported in HF patients with normal sodium levels than in HF patients with lower sodium levels [17], we did not find a correlation between sodium levels and BI scores in the univariate analysis in this study (data not shown).

A limitation of our study was that it was open-label, so patients and physicians knew the study purpose and which patients were in each group. We tried to advise all patients to a similar degree to minimize bed rest in an effort to minimize bias. Other factors, such as the duration of bladder catheterization and interventions for cardiac rehabilitation, are thought to affect outcomes in HF patients; but in this study, there was no significant difference in these factors between the groups. Additionally, release from the infusion line may have prompted an increase in the amount and intensity of exercise in cardiac rehabilitation, which may have improved outcomes, although we did not directly assess this association. The severity of HF may affect functional decline during hospitalization. Because patients with refractory end-stage HF and/or cardiogenic shock frequently need continuous infusion of drugs, including inotropic agents and vasopressors, these patients were excluded from this study; the HF severity was characterized as mild to moderate in all participants. There were no differences in any parameter indicating HF severity, including NYHA symptom class, estimated right ventricular systolic pressure, and serum BNP level. The BI score may have been affected by whether the infusion line was still connected to the patient at the time of the day 10 assessment, although information regarding a patient’s functional status was obtained by interviewing the patient and/or an accompanying family member. In addition, although previous studies have shown that the ADL score at discharge is strongly associated with 2-year mortality [18], the long-term effect of early disconnection of the infusion line on physical function and mortality was not assessed in this study. Further studies are needed.

In conclusion, our study showed that an early switch from continuous IV infusion to OM prevented functional decline in patients hospitalized for HF, without increasing hospital costs. Clinical intervention programs such as ours, may improve patient outcomes.

## Supporting Information

S1 Fig Changes in variables after 10 days of hospitalization. Variables in Table 2 are graphically represented. BI indicates Barthel Index; FIM, Functional Independence Measure; SF-36, 36-item Short-Form General Health Survey; BW, body weight; BNP, B-type natriuretic peptide; eRVPs, estimated right ventricular systolic pressure. *p<0.05, #*p*<0.001.
(TIF)

S1 Table BI individual components. Values are mean ± SD. CI indicates confidence interval; BI, Barthel Index.
(DOCX)

S1 File Study Protocol (Japanese). (DOCX)

S2 File Study Protocol (English translation). (DOCX)

S3 File CONSORT checklist. (DOC)
