# Supplementary figures and images for: Impact of Oral Treatment on Physical Function in Older Patients Hospitalized for Heart Failure: A Randomized Clinical Trial
Source: PLoS One. 2016 Dec 13;11(12):e0167933. doi: 10.1371/journal.pone.0167933 (PMC5154528; doi:10.1371/journal.pone.0167933)

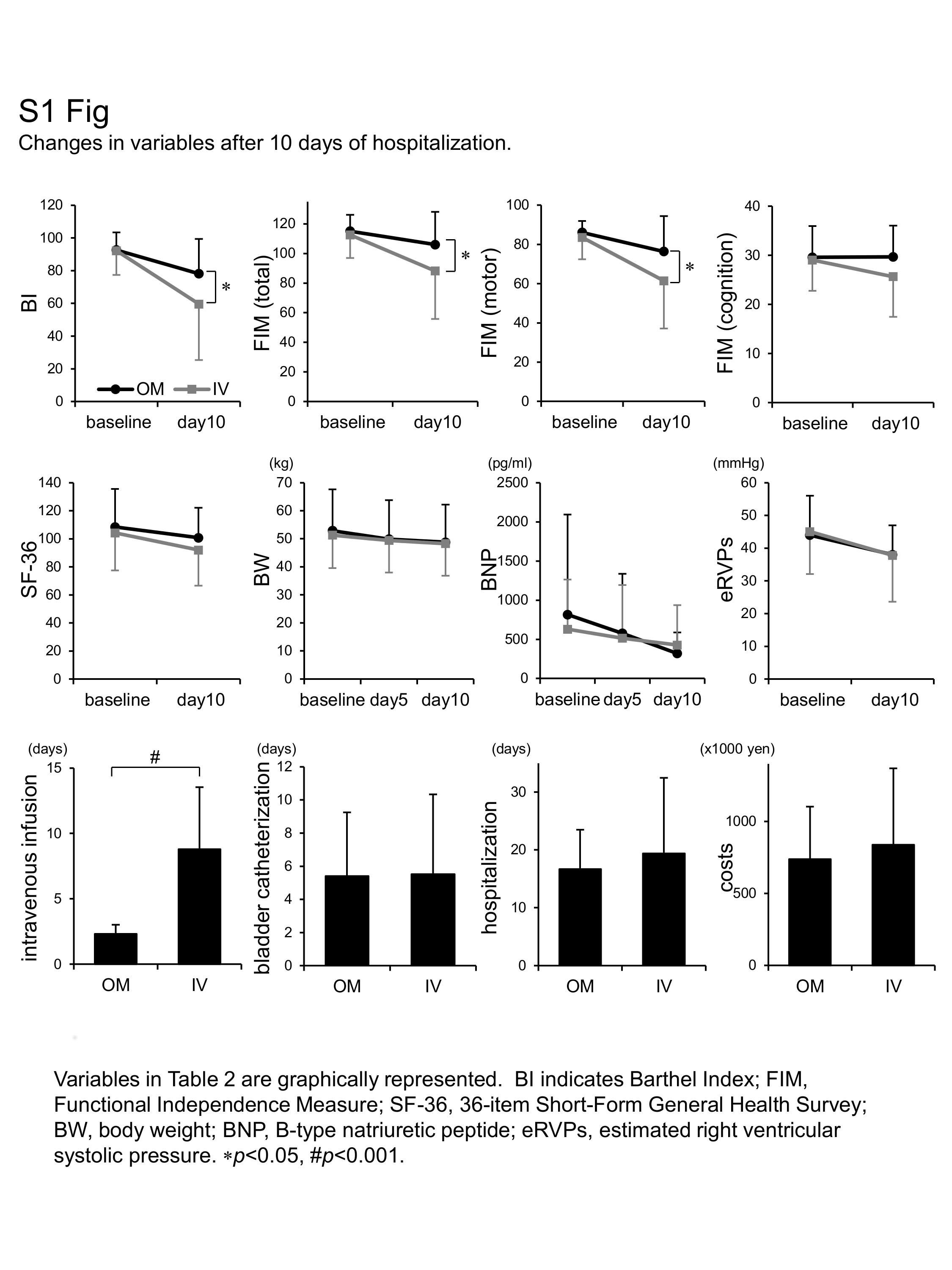

Supplement: S1 Fig — Variables in Table 2 are graphically represented. BI indicates Barthel Index; FIM, Functional Independence Measure; SF-36, 36-item Short-Form General Health Survey; BW, body weight; BNP, B-type natriuretic peptide; eRVPs, estimated right ventricular systolic pressure. *p<0.05, #p<0.001. (TIF) [file pone.0167933.s001.tif]
