# Supplementary material for: Impact of Oral Treatment on Physical Function in Older Patients Hospitalized for Heart Failure: A Randomized Clinical Trial
Source: PLoS One. 2016 Dec 13;11(12):e0167933. doi: 10.1371/journal.pone.0167933 (PMC5154528; doi:10.1371/journal.pone.0167933)
Supplement: S1 Table — Values are mean ± SD. CI indicates confidence interval; BI, Barthel Index. (DOCX) [file pone.0167933.s002.docx]

**S1 Table.** BI individual components

|  | OM group | IV group | OM | vs IV | OM vs IV |
| --- | --- | --- | --- | --- | --- |
|  | (n=25) | (n=25) | (95 | %CI) | p value |
| Feeding (0-10) |  |  |  |  |  |
| baseline | 9.8±1.0 | 9.8±1.0 | 0.0 | (-0.6 to 0.6) | 1.00 |
| day 10 | 9.4±1.7 | 8.5±2.8 | 0.9 | (-0.5 to 2.2) | 0.19 |
| Transfers (0-15) |  |  |  |  |  |
| baseline | 14.4±1.7 | 14.4±3.0 | 0.0 | (-1.4 to 1.4) | 1.00 |
| day 10 | 12.9±3.3 | 9.3±5.7 | 3.6 | (0.9 to 6.3) | 0.01 |
| Grooming (0-5) |  |  |  |  |  |
| baseline | 5.0±0.0 | 4.8±1.0 | 0.2 | (-0.2 to 0.6) | 0.32 |
| day 10 | 4.4±1.7 | 3.0±2.5 | 1.3 | (0.1 to 2.6) | 0.04 |
| Toilet use (1-10) |  |  |  |  |  |
| baseline | 9.6±2.0 | 9.8±1.0 | -0.2 | (-1.1 to 0.7) | 0.66 |
| day 10 | 8.8±2.7 | 6.7±4.2 | 2.0 | (-0.0 to 4.1) | 0.05 |
| Bathing (0-5) |  |  |  |  |  |
| baseline | 4.2±1.9 | 4.2±1.9 | 0.0 | (-1.1 to 1.1) | 1.00 |
| day 10 | 2.3±2.5 | 1.5±2.4 | 0.8 | (-0.7 to 2.2) | 0.29 |
| Ambulation (0-15) |  |  |  |  |  |
| baseline | 14.0±2.0 | 13.2±3.5 | 0.8 | (-0.8 to 2.4) | 0.33 |
| day 10 | 10.2±5.0 | 7.8±6.7 | 2.4 | (-1.1 to 5.8) | 0.17 |
| Stairs (0-10) |  |  |  |  |  |
| baseline | 7.6±3.3 | 8.0±3.5 | -0.4 | (-2.3 to 1.5) | 0.68 |
| day 10 | 4.0±4.4 | 3.3±4.4 | 0.7 | (-1.9 to 3.3) | 0.59 |
| Dressing (0-10) |  |  |  |  |  |
| baseline | 9.8±1.0 | 9.2±2.4 | 0.6 | (-0.4 to 1.6) | 0.25 |
| day 10 | 7.9±2.9 | 5.9±4.2 | 2.0 | (-0.1 to 4.2) | 0.06 |
| Bowels (0-10) |  |  |  |  |  |
| baseline | 9.2±1.9 | 9.6±1.4 | -0.4 | (-1.3 to 0.5) | 0.39 |
| day 10 | 9.6±1.4 | 7.2±4.2 | 2.4 | (0.6 to 4.2) | 0.01 |
| Bladder (0-10) |  |  |  |  |  |
| baseline | 9.2±1.9 | 9.4±1.7 | -0.2 | (-1.2 to 0.8) | 0.69 |
| day 10 | 9.2±2.4 | 6.7±4.4 | 2.4 | (0.3 to 4.5) | 0.02 |

Values are mean ± SD. CI indicates confidence interval; BI, Barthel Index.
