# Supplementary material for: Impact of Oral Treatment on Physical Function in Older Patients Hospitalized for Heart Failure: A Randomized Clinical Trial
Source: PLoS One. 2016 Dec 13;11(12):e0167933. doi: 10.1371/journal.pone.0167933 (PMC5154528; doi:10.1371/journal.pone.0167933)
Supplement: S1 File — (DOCX) [file pone.0167933.s003.docx]

様式―１

受付番号：

平成　　25　年　12　月　2　日

東京警察病院　病院長　殿

科名・職名　　　　循環器科・部長

研究責任者名　　　　笠尾　昌史　　 印

**臨床研究審査申請書**

下記臨床研究の実施について、申請いたします。

記

| １　研究課題名  急性期心不全患者に対する、トルバプタンを含む経口薬管理とルート接続管理によるADL／QOLへの影響に関する研究 |
| --- |
| **２　研究計画**  （１）背景・意義  慢性心不全のうっ血や浮腫のコントロールのために利尿薬は欠かせない治療薬である。しかしながら、ループ利尿薬などの従来型利尿薬は主に塩類排泄型であり、低ナトリウム血症、低カリウム血症などの血清電解質低下１）や、降圧作用２）によるめまい、ふらつきの発現、腎臓への過剰な負荷も懸念される。したがって、塩類排泄型利尿薬を増量、追加出来ないような体液貯留の患者に対しては、新しい作用機序の利尿薬が求められてきた。  2010年12月に「ループ利尿薬などの他の利尿薬で効果不十分な心不全における体液貯留」を効能・効果として塩類排泄を増加させず、水のみを排泄するバソプレシンV2受容体拮抗薬トルバプタン（商品名：サムスカ）が新たな利尿薬として承認された。心不全における体液貯留を対象とした国内第Ⅲ相試験３）では、ループ利尿薬（単独、サイアザイド系利尿薬の併用、抗アルドステロン薬の併用）による治療中の患者を対象とし、1週間の投与期間において体液貯留の改善が示されると共に、血清ナトリウム、血清カリウム等の電解質、血圧・脈拍数、腎機能に対して大きな影響を与えない事が示された。  さらにトルバプタンの有効性については、学会等で多く報告されているが、ADLに与える影響を検討した報告が少ない。  入院患者における、離床時期が患者のADL／QOLに与える影響も報告されており、経口薬管理により急性期心不全患者の早期離床を促し、ADL／QOLを維持することが可能ならば、今後の治療に有用である。  （２）目的  急性期心不全治療において、水利尿薬トルバプタンを含めた経口薬による管理が、患者の離床時期を早め、ADL／QOLの維持にも寄与する可能性を検証する  （３）方法　（①割付の有無　②通常の診療範囲を超える医療行為の内容（侵襲性や試料採取等）について記載すること）  通常の診療範囲内での医療行為にて検証する。  入院初日：初期治療（酸素化、安静、点滴治療）、患者評価・登録  2日目：同意取得、割り付け  同意取得ができた患者を3日目までに、割り付け先の治療を開始する。  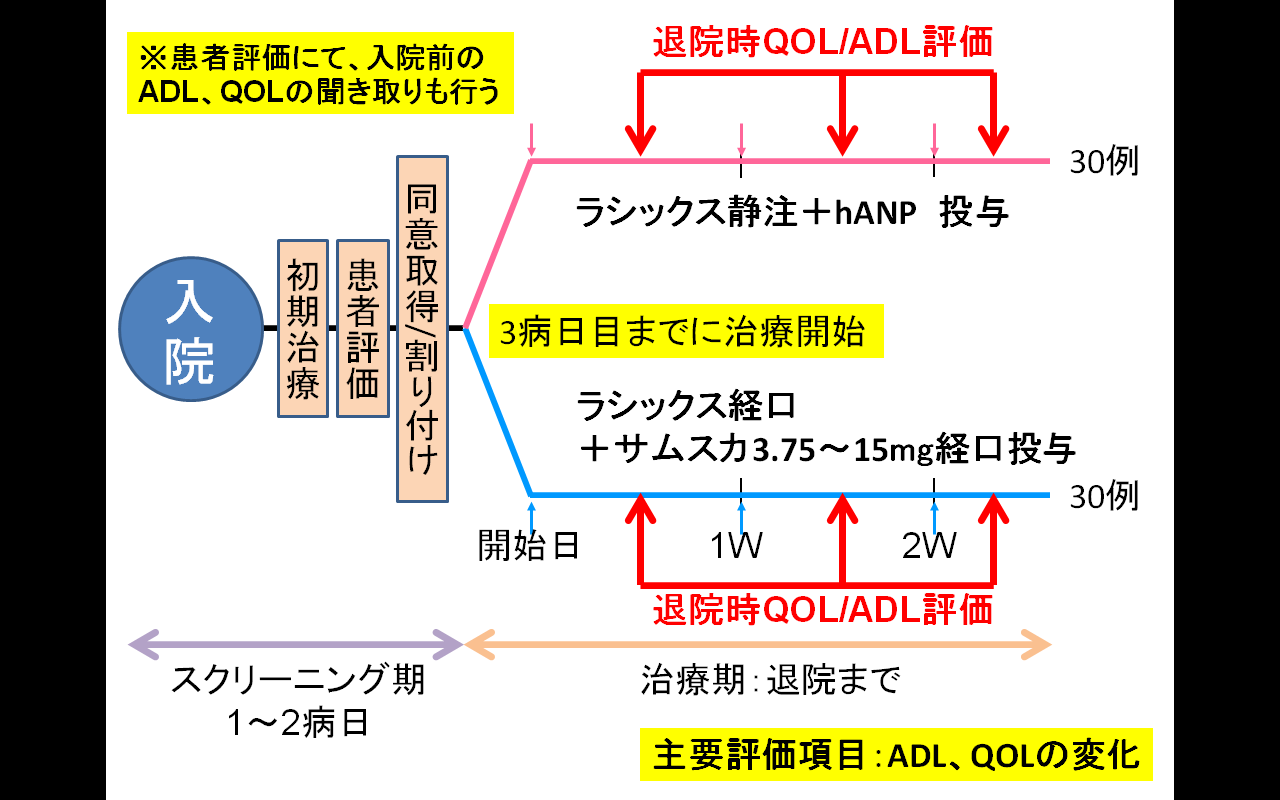  **【選択基準】**  ・患者本人の自由意志による文書同意が得られた患者  ・同意取得時点で20歳以上の患者  ・非代償性心不全患者、もしくは慢性心不全患者の急性増悪で体液貯留を認める  ・以下症状を一つ以上有する  　　呼吸困難／起坐呼吸／下肢浮腫  ・以下徴候を一つ以上有する  　　ラ音／末梢性浮腫／腹水／肺うっ血  **【除外基準】**  ・急性心筋梗塞  ・入院時挿管が必要な患者  ・ショック：sBP≦90mmHg  ・酸素投与下にて動脈血酸素分圧（PaO2）≦60Torr、もしくは動脈酸素飽和度（SpO2）≦90%  ・口渇を訴え、自ら飲水することが出来ない患者  ・維持透析を受けている、もしくは無尿の患者  ・妊婦又は妊娠している可能性のある婦人  ・脱水症状のある患者  ・入院時から既にトルバプタンが投与されている患者  ・担当医師が不適当と判断した患者  **【主要評価項目】**  ・ADLの変化（Barthel Index）  **【副次的評価項目】**  ・ADLの変化（FIM スコア）  ・QOLの変化（SF-36）  ・入院期間  ・尿量  ・飲水量  **【調査項目・検査項目】**  １）患者背景  性別、生年月日、身長、体重、合併症（高血圧、狭心症、糖尿病など）、既往歴、基礎疾患（虚血性心疾患、心筋症、弁膜疾患、高血圧性疾患、不整脈など）、心不全病型（右心不全、左心不全、両心不全）、NYHA重症度分類  ２）併用薬の内容・用量  ３）身体所見（肺うっ血、下肢浮腫、頸静脈怒張、胸水）  ４）血液検査  血算、BUN、Cr、尿酸、Na、K、血漿浸透圧、BNP  ５）尿検査  尿量、UN、Cr、Na、K、尿浸透圧  ６）心エコー  【評価スケジュール】   \| 入院 \| \| 1日目 \| 5日目 \| 10日目または退院時 \| \| --- \| --- \| --- \| --- \| --- \| \| 尿量 \| \|  \|  \|  \| \| 飲水量 \| \|  \|  \|  \| \| 体重 \| \|  \|  \|  \| \| 血圧・心拍数 \| \|  \|  \|  \| \| NYHA \| \| ● \| ● \| ● \| \| 血液検査 \| 一般 \| ● \| ● \| ● \| \| 血清浸透圧 \| ● \| ● \| ● \| \| BNP \| ● \| ● \| ● \| \| 心エコー \| \| ● \|  \| ● \| \| 呼吸困難感 \| \| ● \| ● \| ● \| \| 浮腫 \| \| ● \| ● \| ● \| \| ADL \| \| ● \|  \| ● \| \| QOL \| \| ● \|  \| ● \|   ●：原則トルバプタン、hANP投与前に測定する。  **【被験者の試験参加予定期間】**  試験の参加期間は、退院までとする。  **【試験薬の用法・用量】**  トルバプタンは、原則、3.75～15mgを1日1回投与し、試験担当医師の判断にて適宜増減する。  **【併用薬】**  併用薬に関しては特に制限を設けないが、サムスカ添付文書に記載の「併用注意薬」（下記参照）を使用の際は、安全面に特に配慮する。  ＜サムスカ併用注意薬＞   \| 薬剤名等 \| 臨床症状・措置方法 \| 機序・危険因子 \| \| --- \| --- \| --- \| \| CYP3A4阻害作用を有す る薬剤  ケトコナゾール（経口剤：国内未発売）、イトラコナゾール、クラリスロマイシン等 グレープフルーツジュース \| 代謝酵素の阻害により、本剤の作用が増強するおそれがある。 \| 本剤の代謝酵素であるCYP3A4を阻害 し、本剤の血漿中濃度を上昇させる。 \| \| CYP3A4誘導作用を有す る薬剤  リファンピシン等 セイヨウオトギリソウ（St.John's Wort、セントジョーンズワート）含有食品 \| 代謝酵素の誘導により、本剤の作用が減弱するおそれがあるので、本剤投与時はこれらの薬剤及び食品を摂取しないことが望ましい。 \| 本剤の代謝酵素であるCYP3A4を誘導し、本剤の血漿中濃度を低下させる。 \| \| ジゴキシン \| 本剤によりジゴキシンの作用が増強されるおそれがある。 \| 本剤はP糖蛋白を阻害し、ジゴキシンの血漿中濃度を上昇させる。 \| \| P糖蛋白阻害作用を有する薬剤  シクロスポリン等 \| 本剤の作用が増強するおそれがある。 \| これらの薬剤がP糖蛋白を阻害することにより、本剤の排出が抑制されるため血漿中濃度が上昇するおそれがある。 \| \| カリウム製剤 カリウム保持性利尿薬  スピロノラクトン、トリアムテレン等  抗アルドステロン薬  エプレレノン等  アンジオテンシン変換酵素阻害薬  エナラプリルマレイン酸塩等  アンジオテンシンⅡ受容体拮抗薬  ロサルタンカリウム等  レニン阻害薬  アリスキレンフマル酸塩等 \| これらの薬剤と併用する場合、血清カリウム濃度が上昇するおそれがある。 \| 本剤の水利尿作用により循環血漿量の減少を来し、相対的に血清カリウム濃度が上昇するおそれがある。 \| \| バソプレシン誘導体  デスモプレシン酢酸塩水和物等 \| 本剤によりバソプレシン誘導体の止血作用が減弱するおそれがある。 \| 本剤のバソプレシンV2-受容体拮抗作用により、血管内皮細胞からのvon Willebrand因子の放出が抑制されるおそれがある。 \|   **【研究に参加することにより起こりうる危険並びに必然的に伴う心身に対する不快な状態（有害事象）】**  国内で実施された心性浮腫を対象とした臨床試験において、主な副作用は、口渇30.5％、BUN上昇13.1％、血中尿酸上昇9.4％等であった。詳細は添付文書を参照すること。  本研究の実施に伴い、被験者に健康被害が発生した場合は、研究担当者は適切な処置を講じる。また、本研究では市販薬を適応範囲内で使用することから、健康被害に対する補償は、準備しない。健康被害に対しては、被験者の保険診療内で検査や治療等、必要な処置を行う。この点を研究実施施設の倫理委員会の承認を得るとともに、被験者に十分説明し、理解と同意の上で本研究への参加を求めることとする。  **【研究の中止の条件とその対応】**  研究担当者は、以下の事項に該当する場合は、研究実施継続の可否を検討する。  ①被験者の組み入れが困難で、予定症例数に達することが極めて困難であると判断されたとき。  ②予定症例数または予定期間に達する前に、研究の目的が達成されたとき。  ③倫理委員会から実施計画等の変更の指示があり、受入れることが困難と判断されたとき。  研究責任者は、倫理委員会により中止の勧告あるいは指示があった場合は、研究を中止する。また、研究の中止または中断を決定した時は、速やかに学長及び病院長、研究参加者にその理由とともに報告する。中止までに得られた資料（試料）については、シュレッダーによる破棄もしくは医療廃棄物として破棄するなど、適切な方法により破棄する。  **【結果の解析】**  2群間の解析にはスチューデントt-testを用いる。複数の群間の解析はANOVA解析を行い、post-hoc解析としてTurkey-Kramer HSD analysisを用いる。単変量解析および多変量解析はβ±SEを用いる。解析にはJMP(version 11.0)を用い、P値<0.05を有意とする。 |
| **３　研究デザイン**  ①□ 観察研究　　　　　　 ■ 介入研究  ②■ 前向き研究　　　　　 □ 後向き研究  ③■ 生体試料を用いる □　生体試料を用いない（カルテ情報のみ等）  ④□　遺伝子解析あり　　　■　遺伝子解析なし |
| **４　インフォームド・コンセント**  ■ 文書による説明及び文書による同意　（同意説明文書を添付）  □ 口頭説明及びカルテ等への同意の記録  □ 同意不要（ホームページ上で情報を公開）  □　同意不要 |
| **５　個人情報保護・管理方法**   1. 個人情報管理者名   　所属：循環器科　　　職名：部長　　　氏名：笠尾　昌史   1. 個人情報保護の方法   　　□連結不可能匿名化（個人を識別できないように、その人と新たに付された符号又は番号の対応表を残さない方法（無名化））  　　■連結可能匿名化　（必要な場合に個人を識別できるように、その人と新たに付された符号又は番号の対応表を残す方法（コード化））  　　□その他（   1. 個人情報の管理方法   　　□施錠可能な保管庫  　　□ファイルのパスワード管理  　　■ファイルを施設内のみで管理  　　□その他（ |
| **６　共同研究の有無**  　　■当院単独の研究  　　□多施設共同研究　（主たる研究組織・機関名：　　　　　　　　　　　　　　　　　　研究代表者：　　　　　　　） |
| **７　実施予定期間・予定症例数・実施場所**  （１）実施予定期間　　　2014年　1月　1日　～　　2014年　12月　31日  （２）予定症例数　　　　当院における予定例数：　60　例　　　　試験全体での予定例数：　60　例  （３）実施場所　　　　（　　　　　　　　　　　　　　　　　　　　　　　　　　　　　　　　　） |
| **８　費用の出処及び負担　（利益相反の観点及び患者の費用負担について記載すること）**  利益相反：なし  患者の費用負担：なし |
| **９　参考文献（必要に応じて記入すること）** |

※人体から採取された試料を用いない観察研究の場合、様式-2の入力も必要です。

　様式-2は病院ホームページ上で公開することを原則としています。内容については、研究の独創性及び

　知的財産権の保護のために不利益にならない範囲で、具体的に記載して下さい。
