# Supplementary material for: Impact of Oral Treatment on Physical Function in Older Patients Hospitalized for Heart Failure: A Randomized Clinical Trial
Source: PLoS One. 2016 Dec 13;11(12):e0167933. doi: 10.1371/journal.pone.0167933 (PMC5154528; doi:10.1371/journal.pone.0167933)
Supplement: S2 File — (DOCX) [file pone.0167933.s004.docx]

**Study Protocol**

**(1) Background**

The question of whether treatment with continuous intravenous infusion for older patients hospitalized for heart failure causes functional decline has not been addressed in the literature.

**(2) Objectives**

This study aimed to test whether an early switch from intravenous infusion to oral treatment prevents functional decline in patients hospitalized for heart failure.

**(3) Methods**

Patients are initially treated with continuous peripheral intravenous infusion of standard HF drug such as loop diuretics. Then, within two days after admission, eligible patients are randomized in a 1:1 ratio to either continuous intravenous infusion (IV) group or oral medication (OM) group using computer-generated permuted blocks managed by an independent study center, and participants undergo continuation of the intravenous infusion in IV group or switch to the oral medication in OM group. The study protocol complied with the Declaration of Helsinki.

**[Inclusion criteria]**

Eligible patients are 20 years of age or older hospitalized for acute decompensated HF or exacerbation of chronic HF from any cause presenting signs of volume expansion and New York Heart Association class II to IV symptoms.

**[Exclusion criteria]**

Acute myocardial infarction at the time of hospitalization, hemofiltration or dialysis, and systolic arterial blood pressure less than 90 mmHg. Patients who need continuous fluid replacement and/ or inotropic agent are also excluded from this study.

**[Primary End Point]**

The Barthel Index (BI) is assessed as the primary end point at day 10 or the last day before discharge if patients discharged earlier than day 10.

**[Secondary End Points]**

The second end points include Functional Independent Measure (FIM) and its subscales in which both motor and cognitive function are able to be analyzed. Health-related QOL is assessed with the 36-item Short-Form General Health Survey (SF-36v2). Daily steps were measured by pedometers until day 10 to assess amount of activity in hospital. Length of hospital stay, costs of admission, and place where patients discharged from the trial hospital are also assessed.

[**Measurements**]

1) Baseline characteristics

Age, sex, NYHA classification, sBP, dBP, HR, Etiology of HF, Comorbidity.

2) Dose of IV diuretics prior to randomization.

3) Physical examination

4) Plasma/serum variables (Creatinine, eGFR, Hemoglobin, BNP),

5) Urine variables

6) Echocardiogram (Ejection Fraction, eRVPs)

**[Evaluation time course]**

| Day(s) | | 1 | 5 | 10 or discharge |
| --- | --- | --- | --- | --- |
| Urine Volume | |  |  |  |
| Water intake | |  |  |  |
| Body weight | |  |  |  |
| BP HR | |  |  |  |
| NYHA | | ● | ● | ● |
| Blood test | General | ● | ● | ● |
|  | Serum osmotic pressure | ● | ● | ● |
|  | BNP | ● | ● | ● |
| Echocardiogram | | ● |  | ● |
| Dyspnea | | ● | ● | ● |
| Edema | | ● | ● | ● |
| ADL | | ● |  | ● |
| QOL | | ● |  | ● |

**[Duration]**

The intervention is ended at day 10 or discharge if the participant discharges before day 10.

[**Concomitant medication**]

Participants in the both groups are adequately advised by treatment physicians not to keep the bed rest and were provided with salt-restricted meal (6 gram daily). Fluid intake is restricted to less than 1000 ml daily, except for the cases in which fluid-restriction is cancelled during tolvaptan usage according to its prescribing information. All patients receive standard HF therapy and non-drug treatment such as of bladder catheterization and cardiac rehabilitation program, all of which are employed at the discretion of the treating physician.

[**Cancelation of intervention**]

For the sake of safety, the continuous intravenous infusion is undertaken in the OM group after randomization by the treating physicians’ discretion, in case of such as dehydration and inadequate improvement of HF. Based on intention-to-treat principle, all participants allocated to each group are analyzed.

[**Statistical analysis**]

Continuous variables are presented as mean ± SD or median with interquartile range as appropriate. Comparison between the two groups is performed with Student’s t-test, and comparison between multiple groups are performed with ANOVA followed by Turkey-Kramer HSD analysis. Categorical variables are presented as frequency (percentage) and compared with the Fisher's extract or the chi-square test. Differences in BI scores across daily steps are tested with P for trend obtained from ANOVA. Multivariate analysis is used to assess the independent risk of drug administration route on the clinical end point. The data of univariate and multivariate analysis are presented as β±SE. Analyses are conducted with JMP (version 11.0). All tests of significance are two-tailed, and a P value of less than 0.05 is considered to indicate statistical significance.

**(4) Study design**

1. Interventional study

2. Prospective study

**(5) Informed consent**

All patients provided written informed consent before being enrolled.
